# DNA ploidy may be a prognostic marker in stage I and II serous adenocarcinoma of the endometrium

**DOI:** 10.1007/s00428-012-1275-2

**Published:** 2012-07-24

**Authors:** Manohar Pradhan, Ben Davidson, Vera Maria Abeler, Håvard Emil Danielsen, Claes Göran Tropé, Gunnar Balle Kristensen, Björn Åke Risberg

**Affiliations:** 1 Institute for Medical Informatics, Oslo University Hospital, Oslo, Norway; 2 Department of Pathology, Oslo University Hospital, Oslo, Norway; 3 Center for Cancer Biomedicine, University of Oslo, Oslo, Norway; 4 The Medical Faculty, University of Oslo, Oslo, Norway; 5 Department of Informatics, University of Oslo, Oslo, Norway; 6 Department of Gynecological Oncology, Oslo University Hospital, Oslo, Norway; 7 Department of Pathology, The Norwegian Radium Hospital, Oslo University Hospital, Montebello, 0310 Oslo, Norway

**Keywords:** Serous adenocarcinoma, Endometrial carcinoma, DNA ploidy, p53, Estrogen receptor, Progesterone receptor

## Abstract

In patients with serous adenocarcinoma (SAC) of the endometrium, we evaluated the prognostic importance of clinicopathological parameters, DNA ploidy, and immunoexpression of p53, estrogen receptor (ER), progesterone receptor (PR), and Ki-67. In a series of 73 stage I and II SAC, DNA ploidy analysis was performed on hysterectomy specimens using DNA image cytometry. Immunohistochemical analysis of p53, ER, PR, and Ki-67 expression was additionally performed. In the review of the histological slides by three gynecologic pathologists, the presence of a serous component was not agreed upon in 17 (23 %) cases. The remaining 56 cases, consisting of pure SAC or SAC mixed with endometrioid adenocarcinoma, were further analyzed. Tumor recurrence was observed in 14 patients, and 28 patients died during the follow-up period. Patients with diploid (*n* = 19), aneuploid (*n* = 29), and tetraploid (*n* = 8) tumor had 5-year recurrence rates of 10, 38, and 53 %, respectively (*p* = 0.09). A DNA ploidy parameter, 5c exceeding rate, was found to be a prognostic marker for recurrence (*p* = 0.03), progression-free survival (*p* < 0.01), and overall survival (*p* = 0.02). Immunoexpression of p53, ER, PR, and Ki-67 did not have prognostic value, and the same was true for FIGO stage, lymphovascular invasion, the extent of myometrial invasion, and lymphadenectomy. The histological diagnosis of SAC may be difficult in some cases. Established clinicopathological parameters do not seem to be strong prognosticators in stage I and II disease. A DNA ploidy parameter, 5c exceeding rate, may be a prognostic marker in this patient group and should be further validated in larger series.

## Introduction

Endometrial carcinoma (EC), the most common gynecological malignancy in the Western world, is considered to be a relatively less aggressive tumor with a good prognosis. Nearly 30 years ago, Lauchlan and Hendrickson et al. recognized a distinct histological type, serous adenocarcinoma (SAC), which has a more aggressive behavior [1, 2]. SAC is a rare tumor accounting for 4–10 % of all EC [3, 4]. SAC usually arises from atrophic endometrium and therefore occurs in relatively older women [3, 5]. SAC differs from endometrioid adenocarcinoma (EAC) in a number of respects; estrogen receptor (ER) and progesterone receptor (PR), usually present in normal endometrium and EAC, are less frequently expressed in SAC [6]. The mutation of *TP53*, coding for the tumor suppressor p53, is considered to be an early event in SAC as the mutation has been demonstrated in intraepithelial carcinoma [7]. Ki-67, a nuclear protein associated with cell proliferation, is more extensively expressed in SAC compared to EAC [8]. In contrast to EAC, SAC has not been found to be associated with *PTEN* and *KRAS* mutation or microsatellite instability [9]. Recent gene expression studies have demonstrated different profiles in SAC compared to EAC grade 1 and 2 [10]. SAC is associated with DNA aneuploidy, and most of the tumors show a DNA index >1.60 and a higher 5c exceeding rate (5c ExR) than EAC [11].

The incidence of recurrence after primary therapy is higher in SAC than EAC [12, 13]. Furthermore, SAC is more often diagnosed at advanced stage compared to EAC [3]. In spite of its rarity, SAC has been shown to be responsible for 39 % of all EC-related deaths [4]. However, in an accumulated multinational series of 8,033 EC patients, SAC was found in 323 cases (4 %) with 80 % overall 5-year survival in stage I patients [3]. This indicates that a substantial number of SAC patients have a good prognosis, but reliable prognostic indicators are lacking. In the present study, we evaluated the prognostic role of clinicopathological parameters, DNA ploidy, and immunohistochemical markers in stage I and II SAC.

## Materials and methods

This is a study of consecutive patients with SAC of the endometrium referred to the Norwegian Radium Hospital from October 1998 to December 2007. Altogether, 215 patients were diagnosed with SAC in the period, out of which 73 stage I and II cases were available for DNA ploidy and histological review in hysterectomy specimen. The specimens were retrieved from the archives of the Department of Pathology. Data regarding FIGO stage (2009), the extent of myometrial invasion, and lymphovascular invasion (LVI) were retrieved from pathological reports. Clinical data were provided by the Department of Gynecologic Oncology. Data on patient death were acquired from the death register of the Central Bureau of Statistics, which is based on the transmitted death certificates by the patient’s physician. Simple and radical hysterectomies were performed in 60 and 13 cases, respectively. In 29 patients, infracolic omentectomy was performed. Both pelvic and paraaortic lymphadenectomy were done in 16 cases and only pelvic lymphadenectomy in 26 cases. Twenty-three patients received adjuvant chemotherapy, ten patients received radiotherapy, and five patients received both. Patients with recurrence were treated with chemotherapy (six cases), radiotherapy (five cases), hormonal therapy (one case), both chemotherapy and radiotherapy (two cases), chemotherapy and antihormonal treatment (one case), and all three therapeutic modalities (two cases). Ethical approval for the study was obtained from the Regional Committee for Medical and Health Research Ethics, Southeastern Norway.

### Histologic review

All H&E slides from the area from which DNA ploidy and immunohistochemical analysis were performed were independently reviewed by three experienced gynecological pathologists (BD, BR, and VA). The World Health Organization recommendation [14] was used to classify the tumors. Pure SAC and SAC mixed with EAC were entered in the study. Discrepant cases were discussed at a consensus session.

### DNA image cytometry

Using thick 50-μm sections from paraffin-embedded blocks from hysterectomy specimens, a monolayer of cells was prepared on a slide. After staining with the Feulgen method, nuclear DNA content was measured indirectly using Ploidy Work Station (Room4, Crowborough, UK). DNA ploidy histograms were created using PWS classifier (Room4, Crowborough, UK). The histograms were classified as diploid, aneuploid (DNA index, DI, 1.06–1.89 and >2.10), tetraploid (DI 1.90–2.10), and polyploid. DNA ploidy-related parameters such as DI, 5c ExR, and 9c exceeding rate (9c ExR) of the tumors were also noted. A detailed description of the procedure, DNA content measurement, and histogram classification criteria are given elsewhere [11].

### Immunohistochemistry

From the formalin-fixed, paraffin-embedded tissue blocks of the hysterectomy specimen, 4–5-μm thick sections were cut and stained using a Dako Autostainer. Sections were deparaffinized, and epitopes were unmasked using Dako PT link with high pH buffer for 20 min. Endogenous peroxidase was blocked using EnVision FLEX Peroxidase-Blocking Reagent. The sections were incubated with primary mouse monoclonal antibodies against p53 (1:5,000, Santa Cruz Biotechnology, Inc., Santa Cruz, CA), ER (1:200, Novocastra Laboratories Ltd, Newcastle-upon-Tyne, UK), PR (1:300, Novocastra Laboratories Ltd.), and Ki-67 (1:200, DakoCytomation, Glostrup, Denmark) for 30 min. EnVision FLEX+ Mouse (LINKER) was applied for 15 min for signal amplification. After applying Dako EnVision FLEX + HRP for 30 min, diluted DAB chromogen was applied for 10 min. The slides were counterstained with hematoxylin and mounted. Relevant positive and negative controls were used with each antibody, with satisfactory results. In mixed tumors, expression was scored only in the serous component. Based on percentage of cells showing immunoexpression, slides were scored as negative (0–10 %), weakly positive (11–50 %), and strongly positive (51–100 %).

### Statistical analysis

Predictive Analytics SoftWare statistics 18 (SPSS Inc., Chicago, IL) was used for statistical analysis. All statistical tests were two-sided, and *p* value <0.05 was considered as statistically significant. Progression-free survival (PFS) was calculated from the hysterectomy date to recurrence or 31 March 2009. Both recurrence and death due to any cause were considered as an event for PFS. Overall survival (OS) was calculated from the date of surgery to death or 31 March 2011 using death due to any cause as an event. For survival analysis, median value was used as cutoff for age, 5c ExR, and 9c ExR. The Kaplan–Meier method was used to calculate the 5-year recurrence rate, PFS, and OS. Log-rank test was used for univariate survival analysis.

## Results

### Histological review

After review and consensus discussion, all three or two of the three pathologists agreed on the SAC diagnosis in 56 cases, which were included in further analyses. We used the reviewed diagnosis as the basis for the present study as it was found to be better in predicting prognosis in EC [15].

### Patient characteristics

The median age of patients with SAC was 73 years (mean, 72; range, 56–89 years). There were 43 patients diagnosed at stage IA, 8 at stage IB, and 5 at stage II (Table 1). There was no myometrial invasion in 10 cases, invasion of less than half the myometrial thickness in 34 cases, and more than half in 12 cases. LVI was present in 17 cases. Recurrence was detected in 14 patients, of whom 2 had recurrence in the vagina, 5 in the pelvis, and 7 outside the pelvis. The majority of recurrences occurred in the first year after operation (eight cases); the earliest detected was after 3 months.

**Table 1:** Recurrence rate, progression-free survival, and overall survival based on clinical and pathological parameters in patients with stage I and II serous adenocarcinoma

Variables	Categories	Number of patients (%)	Number of recurrence (%)	Number of death (%)	5-year recurrence rate ± SE (%)	5-year PFS		5-year OS	
						5-year PFS ± SE (%)	*p* value^a^	5-year OS ± SE (%)	*p* value^a^
Age	<72	28 (50)	6 (21)	9 (32)	26 ± 10	59 ± 11	<0.01	69 ± 10	<0.01
	≥72	28 (50)	8 (29)	19 (68)	32 ± 10	38 ± 11		56 ± 10	
Stage	IA	43 (77)	10 (23)	19 (44)	29 ± 8	52 ± 10	NS	69 ± 8	NS
	IB	8 (14)	1 (13)	5 (63)	17 ± 15	25 ± 20		38 ± 17	
	II	5 (9)	3 (60)	4 (80)	60 ± 22	40 ± 22		40 ± 22	
Myometrial invasion	No invasion	10 (18)	2 (20)	3 (30)	32 ± 21	68 ± 21	NS	90 ± 10	NS
	<Half myometrium	34 (61)	9 (26)	17 (50)	30 ± 9	45 ± 11		60 ± 9	
	>Half myometrium	12 (21)	3 (25)	8 (67)	28 ± 14	38 ± 15		42 ± 14	
Adjuvant therapy	Chemotherapy	19 (34)	6 (32)	8 (42)	40 ± 14	60 ± 14	0.02	78 ± 10	0.04
	Radiotherapy	10 (18)	0	3 (30)	–^b^	79 ± 13		80 ± 13	
	Both	2 (4)	0	0	–^b^	–^b^		–^b^	
	No adjuvant therapy	25 (45)	8 (32)	17 (68)	42 ± 13	22 ± 10		42 ± 10	

*PFS* progression-free survival, *OS* overall survival, *SE* standard error, *NS* not significant

^a^Overall *p* value by log-rank test

^b^No statistics were computed because all cases were censored

### DNA ploidy

The mean and median CVs of the diploid peaks were 3.02 and 2.80, respectively. The mean number of nuclei analyzed was 1,005 (median, 1,192; range, 280–2,546). The majority of tumors (*n* = 29, 52 %) were aneuploid, of which 2 cases had DI ≤ 1.20 and 27 cases had DI > 1.20. Tetraploidy and diploidy were detected in 8 (14 %) and 19 (34 %) tumors, respectively (Table 2). Median 5c ExR was 2.28 (mean, 6.08 %; range, 0–72.82 %). Median 9c ExR was 0.07 (mean, 0.42 %; range, 0–13.76 %).

**Table 2:** Recurrence rate, progression-free survival, and overall survival based on DNA ploidy and immunohistochemical markers in patients with stage I and II serous adenocarcinoma

Variables	Categories	Number of patients (%)	Number of recurrences (%)	Number of death (%)	5-year recurrence rate ± SE (%)	5-year PFS		5-year OS	
						5-year PFS ± SE (%)	*p* value^a^	5-year OS ± SE (%)	*p* value^a^
DNA ploidy	Diploid	19 (34)	2 (11)	7 (37)	10 ± 7	61 ± 13	NS	71 ± 11	NS
	Aneuploid	29 (52)	8 (28)	15 (52)	38 ± 13	43 ± 12		64 ± 9	
	Tetraploid	8 (14)	4 (50)	6 (75)	53 ± 19	38 ± 17		38 ± 17	
5c ExR^b^	<2.28 %	28 (50)	4 (14)	10 (36)	15 ± 7	58 ± 11	<0.01	66 ± 9	0.02
	≥2.28 %	28 (50)	10 (36)	18 (64)	48 ± 13	39 ± 11		59 ± 10	
9c ExR^b^	<0.07 %	27 (48)	5 (19)	12 (44)	28 ± 10	51 ± 11	NS	65 ± 9	NS
	≥0.07 %	29 (52)	9 (31)	16 (55)	33 ± 11	45 ± 12		59 ± 10	
P53	Negative (≤10 %)	11 (21)	3 (27)	6 (55)	73 ± 13	62 ± 15	NS	82 ± 12	NS
	Weak (11–50 %)	8 (15)	0	2 (25)	–^c^	75 ± 15		75 ± 15	
	Strong (51–100 %)	34 (64)	11 (32)	20 (59)	59 ± 11	34 ± 10		49 ± 9	
ER	Negative (≤10 %)	33 (63)	9 (27)	17 (52)	67 ± 10	40 ± 10	NS	54 ± 9	NS
	Weak (11–50 %)	13 (25)	4 (31)	9 (69)	67 ± 14	54 ± 14		69 ± 13	
	Strong (51–100 %)	6 (12)	1 (17)	1 (17)	83 ± 15	83 ± 15		83 ± 15	
PR	Negative (≤10 %)	38 (73)	7 (18)	20 (53)	80 ± 7	50 ± 10	NS	58 ± 8	NS
	Weak (11–50 %)	12 (23)	6 (50)	7 (58)	35 ± 18	31 ± 16		58 ± 14	
	Strong (51–100 %)	2 (4)	0	0	–^c^	–^c^		–^c^	
Ki-67	Negative (≤10 %)	1 (2)	0	1 (100)	–^c^	–^c^	NS	–^c^	NS
	Weak (11–50 %)	21 (46)	9 (43)	16 (76)	51 ± 12	27 ± 11		47 ± 11	
	Strong (51–100 %)	24 (52)	2 (8)	8 (33)	90 ± 7	74 ± 9		75 ± 9	

*PFS* progression-free survival, *OS* overall survival, *SE* standard error, *5c ExR* 5c exceeding rate, *9c ExR* 9c exceeding rate, *ER* estrogen receptor, *PR* progesterone receptor, *NS* not significant

^a^Overall *p* value by log-rank test

^b^Median cutoff was used to group the cases

^c^No statistics were computed because all cases were censored

### Immunohistochemistry

Immunohistochemistry was performed in 53 cases for p53, 52 cases for ER and PR, and 46 cases for Ki-67. The majority of tumors (*n* = 34, 64 %) had strong nuclear expression of p53 in >50 % of cells. ER and PR expression in >50 % of cells was found in six (12 %) and two (4 %) tumors, respectively. Ki-67 score >50 % was seen in 24 (52 %) cases (Table 2).

### Survival analysis

**Fig. 1:**
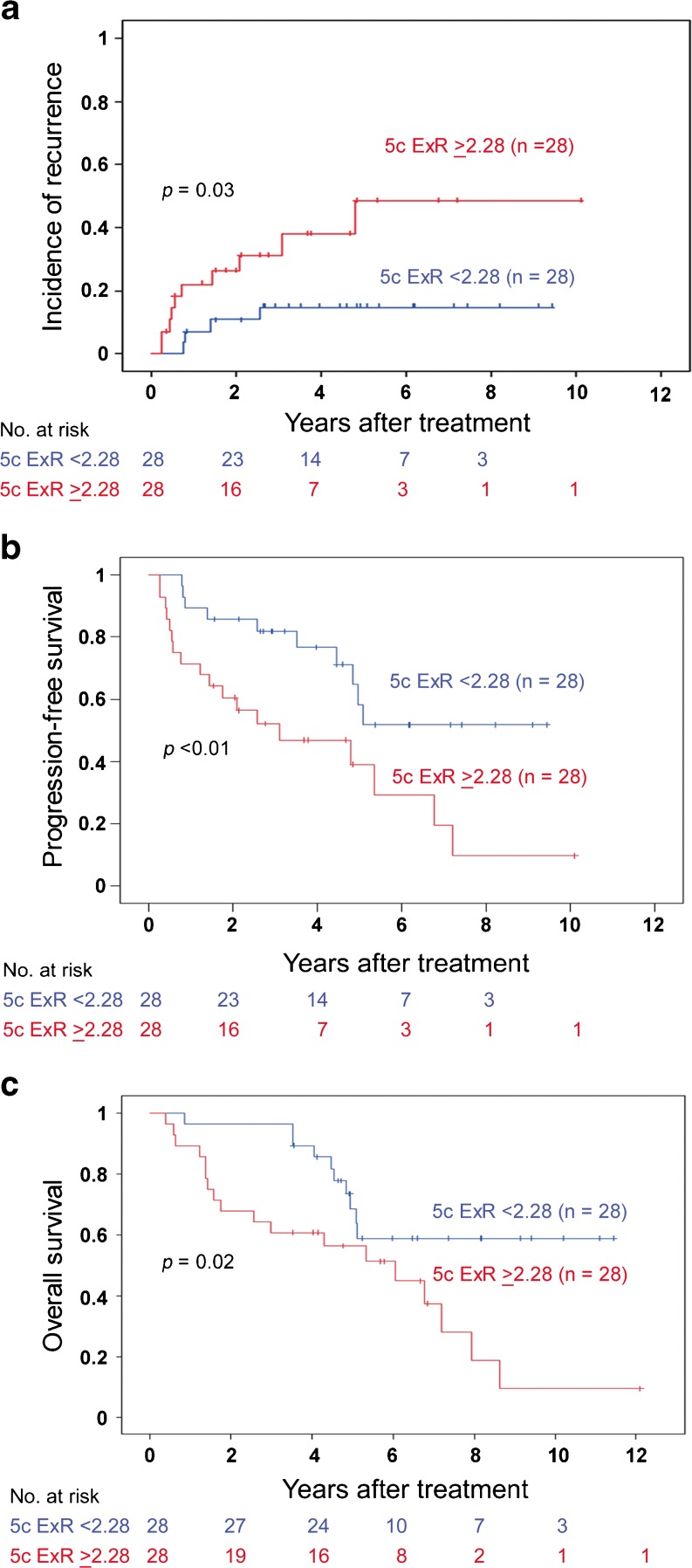
Recurrence rate, progression-free survival, and overall survival based on 5c exceeding rate in patients with serous adenocarcinoma stage I and II

Altogether, 14 patients had recurrent disease, and 28 patients died during the follow-up period. The 5-year PFS and OS for all cases were 48 ± 9 and 62 ± 7 %, respectively. Patients with aneuploid and tetraploid tumors had a higher recurrence rate and lower 5-year PFS and OS compared to patients with diploid tumors (Table 2). DNA ploidy approached, but did not reach statistical significance for recurrence (*p* = 0.09). A DNA ploidy parameter, 5c ExR, was found to be prognostic for 5-year recurrence rate (*p* = 0.03), PFS (*p* < 0.01), and OS (*p* = 0.02; Table 2, Fig. 1). p53, ER, PR, and Ki-67 expression was unrelated to prognosis. Stage, the extent of myometrial invasion, LVI, lymphadenectomy, and the extent of hysterectomy failed to show prognostic significance (Table 1). Shorter 5-year PFS (22 %) and OS (42 %) were observed for patients who did not receive any form of adjuvant therapy. Notably, patients who received radiotherapy as adjuvant treatment had no recurrences.

## Discussion

In this study, we evaluated the prognostic importance of various clinical, pathological, DNA ploidy, and immunohistochemical markers in early-stage SAC. The only significant prognostic marker we found was the 5c ExR, a parameter that can simply be extracted from a DNA ploidy analysis. The explanation is that 5c ExR is high in proliferating tetraploid tumors and aneuploid tumors with high DI. Non-diploid tumors with low S-phase fraction and diploid tumors show low 5c ExR. Therefore, less aggressive non-diploid tumors are separated from highly aggressive tumors. Earlier, Strang et al. reported that 5c ExR is a prognostic parameter in patients with EC [16]. In our previous study, in which exclusively endometrioid carcinoma was analyzed, 5c ExR (1 % as a cutoff) failed to show prognostic importance [13]; however, when the cutoff was changed to 2.28, same as in this series, 5c ExR was a prognostic indicator (unpublished data). Therefore, 5c ExR can be an objective DNA ploidy parameter for patients with endometrial carcinoma. Additionally, 5c ExR has also been found to be a prognostic parameter in other cancers, e.g., non-small cell lung carcinoma, uterine cervical carcinoma, neuroendocrine tumors, and ependymoma [17–20]. There was also a tendency, though not statistically significant, for patients with diploid tumors to have lower recurrence rate and increased overall survival. Kato et al. investigated 30 patients with SAC in all stages and found no association between aneuploidy and survival [21].

**Fig. 2:**
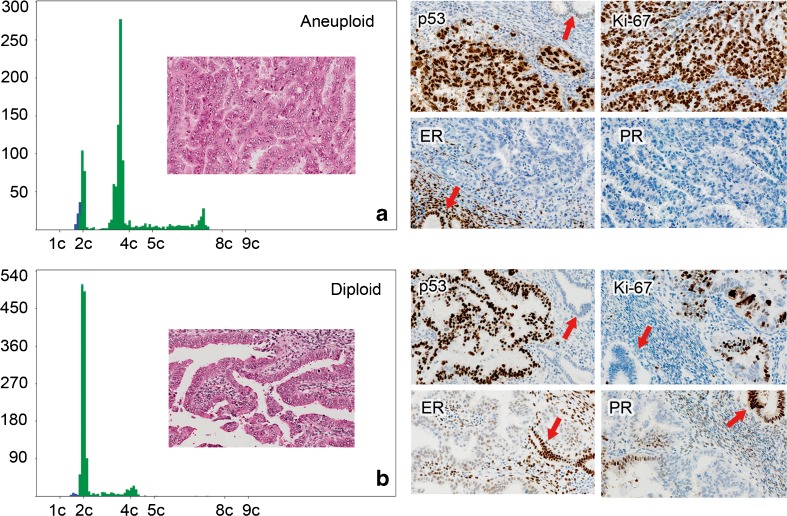
**a** DNA ploidy histogram of an aneuploid serous adenocarcinoma showing high 5c ExR, strongly positive p53, and Ki-67 expression and negative staining for estrogen and progesterone receptors. **b** A diploid serous adenocarcinoma with low 5c ExR showing strong expression of p53 and Ki-67 and weak expression of estrogen and progesterone receptors. Benign glands are marked with *red arrows*

SAC is usually diagnosed based on morphology alone. However, some tumors are difficult to classify due to overlapping features between SAC and EAC [22–24]. We observed lack of agreement in a substantial number of cases at consensus session. This indicates that SAC, even after being a distinct entity for more than 30 years, is not so easy to diagnose morphologically in a certain number of cases. Nordström et al. reported that 12 % of EC were reclassified as SAC following histological review of 266 cases [25]. The differential diagnosis of SAC included both benign and malignant lesions, including EAC with small non-villous papillae, papillary CAC, and malignant mullerian mixed tumors [26]. Therefore, there is a need for ancillary techniques for SAC diagnosis. Many pathologists use overexpression of p53 and Ki-67 together with underexpression of ER and PR as additional diagnostic aids (Fig. 2). Since SAC is associated with aneuploidy with DI >1.60, DNA ploidy might also be a potential parameter supporting the diagnosis [11]. There are reports on markers such as insulin-like growth factor II mRNA-binding protein 3 which may be useful in this differential diagnosis [27].

In EC, stage, myometrial invasion, and LVI are established prognostic parameters [28]. However, these parameters had no prognostic role in our series. Grade and depth of myometrial invasion did not predict prognosis in SAC in an earlier study, and these tumors are currently not graded [21]. Goff et al. analyzed 50 cases of SAC and similarly found no prognostic importance for the depth of myometrial invasion [29].

p53 mutation is considered to be an early event in SAC since it is found in serous intraepithelial carcinoma [7]. Usually, mutation leads to overexpression of mutant p53 protein which can be detected using immunohistochemistry. However, nonsense mutation leads to total absence of protein expression [30]. Although overexpression of p53 has been reported to be a prognostic factor in EC [31], p53 immunostaining was not informative of disease outcome in the present series of SAC. This finding is similar to the observation by Alkushi et al. where p53 overexpression was not a prognostic indicator in patients with SAC but was of prognostic importance in the analysis of patients with low-grade EAC [32]. p53 expression may be helpful in morphologically ambiguous cases due to the higher rate of overexpression in SAC [22]. ER and PR are expressed in normal endometrial tissue, as well as in the majority of EAC, mainly of low grade. In SAC, ER and PR expression was found to be lower than in EAC [33]. ER and PR expression was unrelated to prognosis in our series. High expression of Ki-67, a proliferation marker, was shown to be associated with poor prognosis in patients with EC [31]. However, Ki-67 had no prognostic value in our series of SAC.

In conclusion, established clinicopathological parameters and immunohistochemical markers do not provide prognostic information for patients in SAC stage I and II with high risk of recurrence and death of disease. A DNA ploidy parameter, 5c exceeding rate, may be a prognostic marker in this patient group and should be further validated in larger series.
